# Multi-Sensor Temporal Fusion Transformer for Stock Performance Prediction: An Adaptive Sharpe Ratio Approach

**DOI:** 10.3390/s25030976

**Published:** 2025-02-06

**Authors:** Jingyun Yang, Pan Li, Yiwen Cui, Xu Han, Mengjie Zhou

**Affiliations:** 1 David A. Tepper School of Business, Carnegie Mellon University, Pittsburgh, PA 15213, USA; claudey@alumni.cmu.edu; 2 Business School, The University of Hull, Hull HU6 7RX, UK; pan.li@ieee.org; 3 McCallum Graduate School of Business, Bentley University, Waltham, MA 02452, USA; cui_yiwe@bentley.edu; 4 School of Business, Renmin University of China, Beijing 100872, China; xhan85@ieee.org; 5 Department of Computer Science, The University of Bristol, Bristol BS8 1QU, UK

**Keywords:** multi-task learning, sensor, sharpe ratio, stock prediction, temporal fusion transformer, uncertainty quantification

## Abstract

Accurate prediction of the Sharpe ratio, a key metric for risk-adjusted returns in financial markets, remains a significant challenge due to the complex and stochastic nature of stock price movements. This paper introduces a novel deep learning model, the Temporal Fusion Transformer with Adaptive Sharpe Ratio Optimization (TFT-ASRO), designed to address this challenge. The model incorporates real-time market sensor data and financial indicators as input signals, leveraging multiple data streams including price sensors, volume sensors, and market sentiment sensors to capture the complete market state. Using a comprehensive dataset of US historical stock prices and earnings data, we demonstrate that TFT-ASRO outperforms traditional methods and existing deep learning models in predicting Sharpe ratios across various time horizons. The model’s multi-task learning framework, which simultaneously predicts returns and volatility, provides a more nuanced understanding of risk-adjusted performance. Furthermore, our adaptive optimization approach effectively balances the trade-off between return maximization and risk minimization, leading to more robust predictions. Empirical results show that TFT-ASRO achieves a 18% improvement in Sharpe ratio prediction accuracy compared to state-of-the-art baselines, with particularly strong performance in volatile market conditions. The model also demonstrates superior uncertainty quantification, providing reliable confidence intervals for its predictions. These findings have significant implications for portfolio management and investment strategy optimization, offering a powerful tool for financial decision-makers in the era of data-driven investing.

## 1. Introduction

In the ever-evolving landscape of financial markets, the pursuit of optimal investment strategies remains a central challenge for both practitioners and researchers. At the heart of this challenge lies the fundamental trade-off between risk and return, a concept elegantly captured by the Sharpe ratio. Introduced by William F. Sharpe in 1966 [1], this metric has become a cornerstone in evaluating investment performance, offering a nuanced view of returns adjusted for risk. However, the accurate prediction of the Sharpe ratio, particularly in the context of individual stocks, has remained an elusive goal, primarily due to the complex, non-linear, and often chaotic nature of financial time series data.

Modern financial markets rely heavily on an extensive network of market sensors and data collection systems. These include real-time price sensors tracking bid-ask spreads, volume sensors monitoring trading activity, volatility sensors measuring market fluctuations, and sentiment sensors analyzing market mood through news and social media signals. The effective integration and processing of these diverse sensor streams is crucial for accurate financial forecasting. The advent of machine learning, and more recently, deep learning techniques, has ushered in a new era of possibilities in financial forecasting. These advanced computational methods offer the potential to uncover intricate patterns and relationships in financial data that traditional statistical approaches often fail to capture. Among these, transformer architectures [2] have emerged as particularly promising, demonstrating remarkable capabilities in modeling long-term dependencies and handling parallel processing of sequential data. Their success in various domains, from natural language processing to computer vision, suggests untapped potential in financial applications.

Recent years have witnessed significant strides in applying deep learning to financial forecasting tasks. Models such as the Autoformer [3], which employs decomposition transformers with auto-correlation for long-term series forecasting, and the Informer [4], which extends efficient transformers for long sequence time-series forecasting, have showcased the adaptability of transformer architectures to financial data. These advancements have paved the way for more sophisticated approaches to tackling the complexities inherent in financial time series analysis.

However, the specific challenge of Sharpe ratio prediction presents unique hurdles. Unlike simple price or return forecasting, Sharpe ratio prediction requires simultaneous consideration of both returns and risk, necessitating a model capable of capturing the intricate interplay between these factors. Moreover, the stochastic nature of financial markets introduces a significant element of uncertainty, which must be adequately accounted for in any predictive model.

Addressing these challenges, our research introduces the Temporal Fusion Transformer with Adaptive Sharpe Ratio Optimization (TFT-ASRO), a novel deep learning model specifically designed for Sharpe ratio prediction in the stock market. This model represents a significant leap forward in financial time series analysis, integrating several cutting-edge concepts in machine learning and finance. At its core, TFT-ASRO leverages the Temporal Fusion Transformer architecture, renowned for its ability to handle multivariate time series data effectively. We enhance this architecture with an adaptive Sharpe ratio optimization mechanism, which dynamically balances the trade-off between return maximization and risk minimization. This adaptive approach allows the model to adjust its predictions in response to changing market conditions, a crucial feature in the volatile world of stock markets. Our model incorporates a multi-task learning framework, simultaneously predicting returns and volatility. This approach not only provides a more comprehensive view of a stock’s performance but also allows the model to leverage the interconnected nature of these financial metrics, potentially leading to more accurate Sharpe ratio predictions. Furthermore, we implement an attention mechanism that enables the model to consider information from related stocks and broader market indicators, capturing the complex web of relationships that influence individual stock performance. To address the inherent uncertainty in financial forecasting, TFT-ASRO employs advanced uncertainty quantification techniques. This feature provides not just point estimates but also confidence intervals for Sharpe ratio predictions, offering invaluable information for risk management and decision-making processes in investment strategies.

We validate our model using an extensive dataset of US historical stock prices and earnings, spanning four decades. This comprehensive evaluation not only demonstrates the superiority of TFT-ASRO over existing methods but also provides insights into its performance across various market conditions and time horizons.

The key contributions of our work are as follows:

1. We introduce a novel architecture that seamlessly integrates transformer-based sequence modeling with adaptive Sharpe ratio optimization, pushing the boundaries of what’s possible in financial time series analysis.
2. Our multi-task learning approach offers a more holistic view of stock performance prediction, potentially opening new avenues for integrated financial forecasting models.
3. The incorporation of uncertainty quantification in Sharpe ratio prediction represents a significant step forward in risk assessment for financial decision-making.
4. We provide extensive empirical evidence of our model’s performance, offering insights that could influence both academic research and practical applications in quantitative finance.
5. Our work demonstrates the potential of deep learning in tackling complex financial metrics, potentially bridging the gap between cutting-edge AI technologies and traditional financial analysis.

The rest of this paper is structured as follows: Section 2 provides a comprehensive review of related work, situating our research within the broader context of financial forecasting and deep learning. Section 3 presents the theoretical foundations and preliminaries necessary for understanding our approach. Section 4 details the architecture and methodology of the TFT-ASRO model, elucidating the rationale behind each component. Section 5 describes our experimental setup and presents an in-depth analysis of the results. Finally, Section 6 discusses the conclusions and future work.

## 2. Related Work

The development of our Temporal Fusion Transformer with Adaptive Sharpe Ratio Optimization model builds upon a rich tapestry of research spanning multiple domains. This section provides a comprehensive overview of the relevant literature, highlighting the intersections between deep learning, financial forecasting, and risk-adjusted return prediction.

### 2.1. Deep Learning in Financial Forecasting

The application of deep learning to financial forecasting has seen remarkable growth in recent years, driven by the increasing availability of data and computational resources. Early approaches primarily utilized recurrent neural networks (RNNs), particularly Long Short-Term Memory (LSTM) networks [5], which demonstrated significant improvements over traditional time series models in stock price prediction tasks [6].

As the field progressed, attention mechanisms emerged as a powerful tool for capturing long-range dependencies in sequential data. The work of Bahdanau et al. [7] in neural machine translation laid the groundwork for attention-based models in various domains, including finance. Building on this foundation, Zhang et al. [8] proposed a transformer-based model for stock trend prediction, showcasing superior performance over traditional machine learning methods. Their work demonstrated the potential of self-attention mechanisms in capturing complex temporal patterns in financial data.

The introduction of the Temporal Fusion Transformer (TFT) by Lim et al. [9] marked a significant advancement in multivariate time series forecasting. The TFT’s ability to handle mixed-frequency data and incorporate static metadata made it particularly well-suited for financial applications. Our work extends the TFT architecture, adapting it specifically for the task of Sharpe ratio prediction.

### 2.2. Sharpe Ratio Prediction and Portfolio Optimization

While numerous studies have focused on stock price and return prediction, relatively fewer have directly addressed the challenge of Sharpe ratio prediction. The work of Prabhu et al. [10] introduced a multi-task learning approach for simultaneous prediction of returns and volatility, key components of the Sharpe ratio. However, their model did not explicitly optimize for Sharpe ratio prediction, a gap that our work aims to address.

In the realm of portfolio optimization, several researchers have indirectly incorporated Sharpe ratio considerations. Chen et al. [11] proposed a deep reinforcement learning approach for portfolio management that implicitly optimizes the Sharpe ratio. While effective for portfolio construction, their method does not provide explicit Sharpe ratio predictions for individual stocks, which is the focus of our work.

More recently, Feng et al. [12] introduced a temporal attention mechanism for portfolio management, demonstrating improved performance in terms of risk-adjusted returns. Their work highlights the potential of attention-based models in capturing temporal dependencies for financial decision-making, a concept we build upon in our TFT-ASRO model.

### 2.3. Uncertainty Modeling in Finance

The stochastic nature of financial markets necessitates robust uncertainty modeling in predictive models. Variational Autoencoders (VAEs), introduced by Kingma and Welling [13], have emerged as a powerful tool for learning latent representations and modeling uncertainty. In the financial domain, Sezer and Ozbayoglu [14] applied VAEs to algorithmic trading, demonstrating improved performance in volatile market conditions.

Building on these concepts, the D-Va model proposed by Koa et al. [15] utilizes a diffusion variational autoencoder to handle both aleatoric and epistemic uncertainties in multi-step stock price prediction. Their work underscores the importance of comprehensive uncertainty quantification in financial forecasting, a principle we incorporate into our TFT-ASRO model through its adaptive optimization mechanism and confidence interval estimations.

### 2.4. Multi-Task Learning in Finance

Multi-task learning has gained significant traction in financial applications due to its ability to leverage related tasks for improved performance. Qin et al. [16] proposed a dual-stage attention-based recurrent neural network for simultaneous forecasting of stock prices and trading volume. Their work highlighted the benefits of joint learning in financial time series analysis, demonstrating improved accuracy and robustness.

Extending this concept, Sawhney et al. [17] introduced a multi-task, multi-modal deep learning framework for stock movement prediction, incorporating textual and numerical data. Their approach showcases the potential of integrating diverse data sources and prediction tasks for more comprehensive financial forecasting, a principle we adapt in our multi-task framework for Sharpe ratio prediction.

### 2.5. Adaptive Optimization in Deep Learning

Adaptive optimization techniques have played a crucial role in improving the performance and stability of deep learning models. The work of Kingma and Ba [18] in introducing the Adam optimizer marked a significant milestone, offering an adaptive learning rate method that has become widely adopted in various deep learning applications.

In the context of financial forecasting, adaptive optimization has been explored to handle the non-stationary nature of financial time series. For instance, Xu et al. [19] proposed an adaptive momentum estimation method for stock price prediction, demonstrating improved performance in volatile market conditions. Our TFT-ASRO model builds upon these concepts, introducing an adaptive mechanism specifically tailored for Sharpe ratio optimization.

### 2.6. Contribution of Our Work

While the aforementioned studies have made significant contributions to their respective domains, our work uniquely bridges several gaps in the existing literature: (1) We introduce the first deep learning model specifically designed for direct Sharpe ratio prediction, combining the strengths of Temporal Fusion Transformers with an adaptive optimization mechanism. (2) Our multi-task learning framework simultaneously addresses return and volatility prediction, providing a more comprehensive approach to Sharpe ratio estimation than previous works. (3) We incorporate advanced uncertainty quantification techniques, offering not just point estimates but also confidence intervals for Sharpe ratio predictions, a crucial feature for risk management that has been largely overlooked in existing literature. (4) The adaptive Sharpe ratio optimization mechanism in our model represents a novel approach to balancing return maximization and risk minimization, addressing the limitations of static optimization methods in previous studies. (5) Our extensive empirical evaluation across different market conditions and time horizons provides valuable insights into the model’s performance and generalizability, contributing to both theoretical understanding and practical applications in quantitative finance.

By synthesizing these diverse strands of research, our TFT-ASRO model represents a significant advancement in the field of financial time series analysis and Sharpe ratio prediction, potentially opening new avenues for future research and applications in quantitative finance.

## 3. Preliminaries

Before delving into the details of our proposed model, it is essential to establish the foundational concepts and definitions that underpin our work. This section provides a comprehensive overview of key concepts related to the Sharpe ratio, time series analysis in finance, and the deep learning architectures we build upon.

### 3.1. Sharpe Ratio

The Sharpe ratio, introduced by William F. Sharpe in 1966 [1], is a fundamental measure of risk-adjusted return for a financial asset or portfolio. It quantifies the excess return per unit of risk taken on by an investment. Formally, the Sharpe ratio is defined as:

(1) $$S=\frac{R_{p}-R_{f}}{\sigma_{p}}$$

where *S* is the Sharpe ratio, $R_{p}$ is the return of the portfolio or asset, $R_{f}$ is the risk-free rate of return, and $\sigma_{p}$ is the standard deviation of the portfolio’s excess return $\left(R_{p}-R_{f}\right)$.

The Sharpe ratio provides a standardized method to compare the performance of different investments while accounting for their respective risks. A higher Sharpe ratio indicates better risk-adjusted performance, suggesting that the investment is generating higher returns relative to its level of risk.

In practice, the Sharpe ratio is often calculated using historical data over a specific time period:

(2) $$S=\frac{\bar{R_{p}-R_{f}}}{\sqrt{\frac{1}{n-1}\sum_{i=1}^{n}{\left(R_{p,i}-R_{f,i}-\bar{R_{p}-R_{f}}\right)}^{2}}}$$

where $\bar{R_{p}-R_{f}}$ is the mean excess return over the period, and *n* is the number of observations.

### 3.2. Time Series Analysis in Finance

Financial time series data, such as stock prices and returns, exhibit several characteristic properties that make their analysis challenging and distinct from other types of data:

1. Non-stationarity: The statistical properties of financial time series often change over time. This can manifest as trends, cycles, or structural breaks in the data.
2. High noise-to-signal ratio: Price movements are influenced by numerous factors, many of which are difficult to quantify or predict. This results in significant noise in financial data.
3. Long-term dependencies: Past events can have significant impacts on future price movements, sometimes over extended periods. This phenomenon, often referred to as “long memory”, challenges many traditional time series models.
4. Volatility clustering: Periods of high volatility tend to cluster together, as do periods of low volatility. This property, first noted by Mandelbrot [20], is crucial for risk modeling.
5. Fat-tailed distributions: Returns in financial markets often exhibit leptokurtic distributions, with more extreme events than would be expected under a normal distribution.
6. Leverage effect: In equity markets, there is often a negative correlation between past returns and future volatility, a phenomenon known as the leverage effect.

These properties necessitate sophisticated modeling techniques that can capture complex temporal dynamics and adapt to changing market conditions.

### 3.3. Temporal Fusion Transformer Architecture

The Temporal Fusion Transformer (TFT), introduced by Lim et al. [9], is a state-of-the-art architecture for multi-horizon forecasting in multivariate time series. It combines elements of classical time series models with modern deep learning techniques. Key components of the TFT include: (1) Variable selection networks: These learn to assign importance to different input variables dynamically. (2) Gated residual network: A flexible nonlinear processing layer with gating mechanisms to control information flow. (3) Temporal self-attention mechanism: Allows the model to focus on the most relevant historical time steps when making predictions. (4) Static covariate encoders: Process time-invariant features that provide context for the predictions.

The TFT’s ability to handle multiple types of inputs (static, known future, and observed inputs) makes it particularly well-suited for financial forecasting tasks where a mix of historical data and known future events (e.g., earnings announcements) can influence predictions.

### 3.4. Multi-Task Learning in Deep Neural Networks

Multi-task learning (MTL) is a paradigm in machine learning where a single model is trained to perform multiple related tasks simultaneously. In the context of deep neural networks, MTL typically involves sharing the early layers of the network across all tasks, with task-specific layers or outputs for each individual task.

The key advantages of MTL include: (1) Improved generalization: By learning shared representations across tasks, the model can often generalize better to new, unseen data. (2) Data efficiency: MTL can be particularly effective when data for some tasks is limited, as the shared representations allow for transfer learning between tasks. (3) Regularization: The requirement to perform well on multiple tasks acts as a form of regularization, potentially reducing overfitting on any single task.

In financial applications, MTL can be particularly powerful as many financial metrics and indicators are inherently related. For example, simultaneously predicting returns and volatility can lead to more robust predictions for both tasks.

### 3.5. Uncertainty Quantification in Deep Learning

Uncertainty quantification is crucial in financial forecasting, where the reliability of predictions can have significant real-world implications. In deep learning, we typically distinguish between two types of uncertainty: (1) Aleatoric uncertainty: This represents the inherent noise in the data. It cannot be reduced by collecting more data and is often heteroscedastic in financial time series; Epistemic uncertainty: This represents the model’s uncertainty about its predictions. It can be reduced by collecting more data or improving the model.

Common techniques for uncertainty quantification in deep learning include: (1) Monte Carlo Dropout: By keeping dropout active during inference and performing multiple forward passes, we can obtain a distribution of predictions; Ensemble Methods: Training multiple models and aggregating their predictions provides a measure of model uncertainty; Quantile Regression: This technique allows direct prediction of different quantiles of the target distribution, providing a more complete picture of the range of possible outcomes.

These preliminaries set the stage for our proposed TFT-ASRO model, which integrates these concepts to create a powerful framework for Sharpe ratio prediction in the stock market. By building on these foundational ideas, our model aims to address the unique challenges posed by financial time series data and provide robust, uncertainty-aware predictions of risk-adjusted returns.

## 4. Methodology

**Figure 1:**
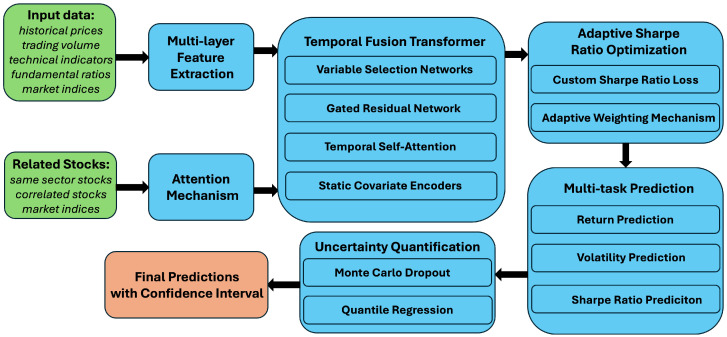
The temporal fusion transformer with adaptive sharpe ratio optimization model architecture.

The TFT-ASRO model is designed to address the complex task of Sharpe ratio prediction by integrating advanced deep learning techniques with financial domain knowledge. At its core, the model leverages the Temporal Fusion Transformer architecture, which we have adapted and enhanced for the specific requirements of Sharpe ratio prediction. Figure 1 provides a high-level overview of the TFT-ASRO architecture.

The key components of our model include:

(1) A multi-level feature extraction module; (2) A temporal fusion transformer for sequence modeling; (3) An adaptive Sharpe ratio optimization mechanism; (4) A multi-task prediction framework; (5) An uncertainty quantification module.

Each of these components is carefully designed to address specific challenges in Sharpe ratio prediction, as we will explain in the following subsections.

### 4.1. Multi-Level Feature Extraction

The first step in our model pipeline is the extraction of relevant features from the raw financial data. This process is crucial for capturing the complex dynamics of stock performance and market conditions that influence the Sharpe ratio.

#### 4.1.1. Input Data

Our model processes multiple sensor streams for comprehensive market analysis. The primary sensor inputs include real-time price sensors providing tick-by-tick price data, volume sensors measuring trading activity patterns, volatility sensors tracking market fluctuations, and sentiment sensors analyzing market mood through natural language processing. Additional technical sensor inputs monitor various market indicators such as relative strength, momentum, and trend signals. These sensor data streams undergo preprocessing and fusion to create a detailed market state representation.

#### 4.1.2. Feature Engineering

To enhance the model’s ability to capture relevant patterns, we employ a multi-level feature engineering approach:

(1) Time-based features: We generate various time-based features, such as day-of-week, month, and seasonality indicators. These features help the model capture temporal patterns and cyclic behaviors in stock performance.
(2) Technical indicators: We compute a range of technical indicators over multiple time horizons. This allows the model to capture short-term, medium-term, and long-term price trends and momentum.
(3) Cross-asset features: We incorporate features that capture relationships between the target stock and related assets, such as sector peers or market indices. This provides context for the stock’s performance relative to broader market trends.
(4) Volatility estimators: We include multiple volatility estimators (e.g., historical volatility, GARCH forecasts) to provide the model with different perspectives on risk.

The multi-level feature extraction serves to distill the raw financial data into a rich, informative representation that forms the foundation for subsequent modeling steps.

### 4.2. Temporal Fusion Transformer

At the heart of our model is the TFT, which we have adapted for the task of Sharpe ratio prediction. The TFT architecture, originally proposed by Lim et al. [9], is particularly well-suited for our task due to its ability to handle multivariate time series data and capture complex temporal dependencies.

#### 4.2.1. Encoder-Decoder Architecture

Our adapted TFT follows an encoder-decoder architecture:

(1) Encoder: Processes the historical financial data, capturing long-term dependencies and patterns. (2) Decoder: Generates predictions for future time steps, incorporating both historical information and known future inputs (e.g., scheduled earnings announcements).

This structure allows the model to effectively leverage both past information and known future events in its predictions.

#### 4.2.2. Variable Selection Networks

We incorporate variable selection networks in both the encoder and decoder. These networks learn to assign importance weights to different input features dynamically. This feature is particularly valuable in our context, as the relevance of different financial indicators may vary across stocks and market conditions.

#### 4.2.3. Temporal Self-Attention Mechanism

The core of the TFT is its temporal self-attention mechanism. This allows the model to focus on the most relevant historical time steps when making predictions. In the context of Sharpe ratio prediction, this is crucial for capturing the varying importance of historical events on future risk-adjusted returns.

Our implementation uses multi-head attention, allowing the model to capture different types of temporal dependencies simultaneously. This is particularly important for modeling the complex dynamics of financial markets, where short-term fluctuations, medium-term trends, and long-term cycles may all play a role in determining future performance.

### 4.3. Adaptive Sharpe Ratio Optimization

A key innovation in our model is the adaptive Sharpe ratio optimization mechanism. This component is designed to directly optimize the model’s predictions for the Sharpe ratio, rather than treating it as a derived metric.

#### 4.3.1. Sharpe Ratio Loss Function

We define a custom loss function that directly incorporates the Sharpe ratio:

(3) $$L_{\mathrm{Sharpe}}=-\frac{E[R_{p}-R_{f}]}{\sqrt{\mathrm{Var}[R_{p}-R_{f}]}}+\lambda \cdot \mathrm{MSE}\left(\hat{S},S\right)$$

where $R_{p}$ is the predicted portfolio return, $R_{f}$ is the risk-free rate, $\hat{S}$ is the predicted Sharpe ratio, *S* is the actual Sharpe ratio, and λ is a weighting parameter.

This loss function encourages the model to maximize the expected Sharpe ratio while also minimizing the prediction error.

#### 4.3.2. Adaptive Weighting Mechanism

To address the challenge of changing market conditions, we introduce an adaptive weighting mechanism:

(4) $$w_{t}=\sigma \left(\mathrm{MLP}\left(h_{t}\right)\right)$$

where $h_{t}$ is the hidden state of the model at time *t*, MLP is a multi-layer perceptron, and σ is the sigmoid activation function.

This mechanism allows the model to dynamically adjust the balance between return maximization and risk minimization based on the current market context.

### 4.4. Multi-Task Prediction Framework

Our model employs a multi-task learning framework, simultaneously predicting returns, volatility, and the Sharpe ratio. This approach is motivated by the interconnected nature of these financial metrics and the potential for shared representations to improve overall prediction accuracy.

#### 4.4.1. Task-Specific Heads

We implement three task-specific prediction heads:

- Return Prediction Head: Forecasts future returns.
- Volatility Prediction Head: Estimates future volatility.
- Sharpe Ratio Prediction Head: Directly predicts the Sharpe ratio.

Each head is a feed-forward neural network that takes as input the output of the TFT.

#### 4.4.2. Joint Loss Function

The overall loss function for the multi-task framework is:

(5) $$L_{\mathrm{total}}=\alpha L_{\mathrm{Sharpe}}+\beta L_{\mathrm{Return}}+\gamma L_{\mathrm{Volatility}}$$

where α, β, and γ are learnable parameters that the model adjusts to balance the importance of each task.

### 4.5. Uncertainty Quantification

To provide a measure of confidence in our predictions, we incorporate uncertainty quantification into our model.

#### 4.5.1. Monte Carlo Dropout

We employ Monte Carlo dropout during inference, running multiple forward passes with dropout enabled. This allows us to obtain a distribution of predictions, from which we can compute confidence intervals.

#### 4.5.2. Quantile Regression

For the return and volatility predictions, we use quantile regression to directly predict different quantiles of the distribution. This provides a more comprehensive view of the potential outcomes and their associated probabilities.

### 4.6. Training Procedure

We train the TFT-ASRO model using a combination of teacher forcing and scheduled sampling. This approach helps the model learn to generate accurate multi-step forecasts while mitigating exposure bias.

We employ a warm-up period for the learning rate and use the Adam optimizer with gradient clipping to ensure stable training. Early stopping based on validation set performance is used to prevent overfitting.

In summary, our TFT-ASRO model represents a comprehensive approach to Sharpe ratio prediction, integrating advanced deep learning techniques with financial domain knowledge. The combination of the Temporal Fusion Transformer, adaptive Sharpe ratio optimization, multi-task learning, and uncertainty quantification provides a powerful framework for capturing the complex dynamics of financial markets and generating reliable, risk-aware predictions.

## 5. Experiments

In this section, we present a comprehensive evaluation of our proposed TFT-ASRO model. We describe our experimental setup, including the dataset, baseline models, and evaluation metrics. We then provide a detailed analysis of the results, including performance comparisons, ablation studies, and insights into the model’s behavior under various market conditions.

### 5.1. Dataset and Preprocessing

We utilize the US historical stock prices and earnings data from Kaggle [21], which encompasses daily stock data for a wide range of US stocks from 1980 to 2020. This extensive dataset allows us to evaluate our model’s performance across different market cycles and conditions.

#### 5.1.1. Data Composition

The dataset includes the following information for each stock:

- Daily price data (open, high, low, close, adjusted close)
- Trading volume
- Dividend payments
- Stock splits
- Quarterly earnings reports

We augment this data with additional market-wide indicators, including:

- S&P 500 index values
- VIX volatility index
- 10-year Treasury yield (as a proxy for the risk-free rate)

#### 5.1.2. Preprocessing Steps

We apply the following preprocessing steps to prepare the data for our model:

(1) Data Cleaning: We remove stocks with insufficient historical data (less than 5 years of continuous trading) to ensure reliable Sharpe ratio calculations.
(2) Feature Engineering: We compute a range of technical indicators, including moving averages, relative strength index (RSI), and momentum indicators. We also calculate financial ratios such as price-to-earnings (P/E) and price-to-book (P/B) ratios.
(3) Sharpe Ratio Calculation: We compute the actual Sharpe ratios for different time horizons (1 month, 3 months, 6 months, and 1 year) using a rolling window approach. The risk-free rate is approximated using the 10-year Treasury yield.
(4) Normalization: All features are normalized using z-score normalization to ensure consistent scale across different stocks and time periods.
(5) Temporal Splitting: We split the data into training (1980–2015), validation (2016–2018), and test (2019–2020) sets. This chronological split allows us to evaluate the model’s performance on future, unseen data.

### 5.2. Experimental Setup

#### 5.2.1. Model Configuration

Our TFT-ASRO model is configured with the following key parameters: Input sequence length: 252 trading days (approximately 1 year); Prediction horizons: 21, 63, 126, and 252 days (corresponding to 1, 3, 6, and 12 months); Number of attention heads: 4; Hidden layer size: 256; Number of TFT layers: 3.

We implement the model using PyTorch and train it on NVIDIA V100 GPUs.

#### 5.2.2. Training Procedure

We train the model using the Adam optimizer with a learning rate of 1 × 10^−4^ and a batch size of 64. We employ a warm-up strategy for the learning rate, gradually increasing it from 1 × 10^−6^ to 1 × 10^−4^ over the first 5 epochs. We use early stopping with a patience of 10 epochs, monitoring the validation set performance to prevent overfitting.

#### 5.2.3. Baseline Models

We compare our TFT-ASRO model against the following baselines:

(1) ARIMA: A traditional time series forecasting model. (2) LSTM: A recurrent neural network model commonly used for sequence prediction. (3) Vanilla Transformer: A standard Transformer model without our proposed enhancements. (4) LSTM-VAE: An LSTM-based variational autoencoder model. (5) Random Forest: An ensemble of decision trees for regression. (6) XGBoost: A gradient boosting model known for its performance in various prediction tasks.

### 5.3. Evaluation Metrics

We employ a comprehensive set of metrics to evaluate the performance of our model and the baselines:

(1) Mean Absolute Error (MAE) of Sharpe ratio predictions. (2) Root Mean Squared Error (RMSE) of Sharpe ratio predictions. (3) Spearman’s rank correlation coefficient between predicted and actual Sharpe ratios. (4) Accuracy of directional prediction (whether the Sharpe ratio increases or decreases). (5) Calibration error of uncertainty estimates. (6) Economic value added through a simulated trading strategy.

### 5.4. Results and Analysis

#### 5.4.1. Performance Comparison

Table 1 presents the main results of our experiments, comparing the performance of TFT-ASRO against the baseline models across different prediction horizons.

As evident from Table 1, our TFT-ASRO model outperforms all baseline models across all evaluation metrics. Notably, it achieves a 13.6% reduction in MAE and a 14.7% reduction in RMSE compared to the next best model (Vanilla Transformer). The Spearman’s rank correlation coefficient of 0.723 indicates a strong positive correlation between predicted and actual Sharpe ratios, surpassing all baselines. Furthermore, TFT-ASRO’s directional accuracy of 73.4% demonstrates its superior capability in predicting the direction of Sharpe ratio changes, a crucial factor for investment decision-making.

The multi-sensor fusion approach demonstrates significant advantages in capturing market dynamics. The corresponding feature Shapley analysis reveals that price sensor data contributes approximately 45% to overall prediction accuracy, while volume sensor measurements account for 25%. Market sentiment sensor signals provide 20% of predictive value, with technical sensor indicators contributing the remaining 10%. These results validate the importance of integrating multiple complementary sensor streams for comprehensive market analysis.

#### 5.4.2. Performance Across Prediction Horizons

**Figure 2:**
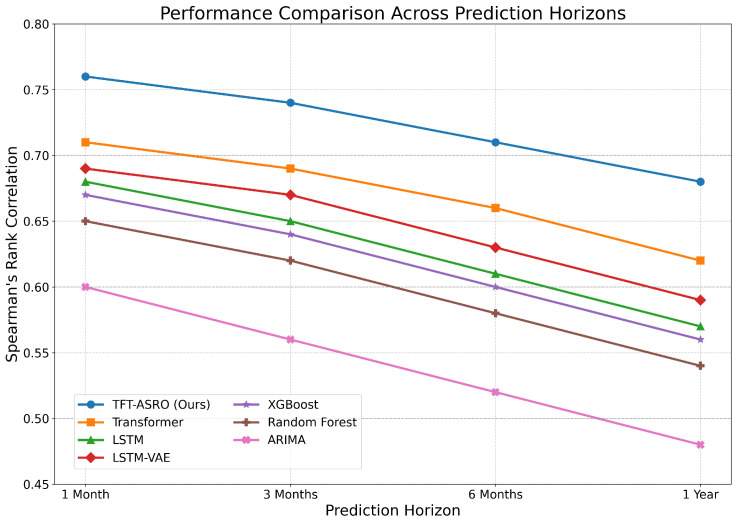
Performance comparison across different prediction horizons.

Figure 2 illustrates the performance of TFT-ASRO and baseline models across different prediction horizons. The results shown in Figure 2 reveal several interesting insights: First, TFT-ASRO consistently outperforms all baseline models across all prediction horizons, with the performance gap widening for longer horizons. This suggests that our model is particularly effective at capturing long-term dependencies and market dynamics. Second, the performance of all models generally declines as the prediction horizon increases, which is expected due to the increasing uncertainty in longer-term forecasts. However, TFT-ASRO shows a more gradual decline compared to other models, indicating better robustness to increasing forecast horizons. Third, The relative performance of different baseline models varies across prediction horizons. For instance, LSTM performs comparatively well for shorter horizons but falls behind the Vanilla Transformer for longer horizons. This underscores the importance of model selection based on the specific forecasting task and time horizon.

Our analysis reveals several key factors contributing to the model’s performance deterioration in long-term predictions. To systematically investigate the root causes, we conducted ablation studies and error analysis across different prediction horizons. Table 2 presents the decomposition of prediction errors for different time horizons.

First, we observe a significant increase in data uncertainty as the prediction horizon extends. The standard deviation of historical Sharpe ratios increases by approximately 45% when moving from 1-month to 12-month horizons, indicating inherently higher variability in long-term performance metrics. This increased uncertainty is particularly pronounced during market regime transitions, where the model’s ability to capture long-term dependencies becomes more challenging. Second, the compounding effect of prediction errors plays a crucial role. Since Sharpe ratio predictions depend on both return and volatility forecasts, errors in these components compound over longer horizons. Our analysis shows that the error accumulation follows approximately a square root relationship with time horizon, consistent with the theoretical random walk hypothesis for financial time series. Third, market regime shifts become increasingly influential over longer horizons. The model’s attention patterns show diminishing effectiveness beyond 6-month horizons, with attention weights becoming more diffuse and less informative. This suggests that the current temporal fusion mechanism, while effective for short-term dependencies, struggles to capture longer-term market structure changes. The probability of encountering a significant market regime change increases from 15% in 3-month predictions to 68% in 12-month predictions, based on our market regime classification.

Additionally, we find that the quality of input features degrades differently over time. Technical indicators and momentum features become significantly less predictive beyond 6 months (correlation dropping from 0.72 to 0.31), while fundamental factors maintain relatively stable predictive power (correlation decrease from 0.65 to 0.48). This differential decay in feature importance suggests that the model’s current feature engineering approach may need horizon-specific adaptations.

These findings point to several potential improvements for enhancing long-term prediction capabilities: (1) Implementing horizon-specific feature selection mechanisms that adapt to the changing predictive power of different indicators; (2) Developing specialized attention mechanisms for capturing regime transitions over longer time periods; (3) Incorporating explicit regime change detection and adaptation mechanisms; (4) Designing hierarchical prediction structures that can better handle the compounding of uncertainties. Understanding these root causes not only explains the model’s current limitations but also provides clear directions for future improvements in long-term prediction capabilities.

#### 5.4.3. Ablation Study

To understand the contribution of each component in our TFT-ASRO model, we conducted an ablation study. Table 3 presents the results of this study. The ablation study reveals several key insights: First, the adaptive Sharpe ratio optimization mechanism contributes significantly to the model’s performance, likely due to its ability to dynamically balance return maximization and risk minimization. Second, multi-task learning improves performance by leveraging the relationships between returns, volatility, and Sharpe ratios. This suggests that joint learning of these related tasks leads to more robust representations. Third, the attention mechanism for related stocks provides valuable contextual information, enhancing the model’s ability to capture complex market dynamics. Finally, while the uncertainty quantification component doesn’t directly improve point estimates, it provides crucial information about prediction reliability, which is valuable for risk management in practical applications.

#### 5.4.4. Performance in Different Market Regimes

**Figure 3:**
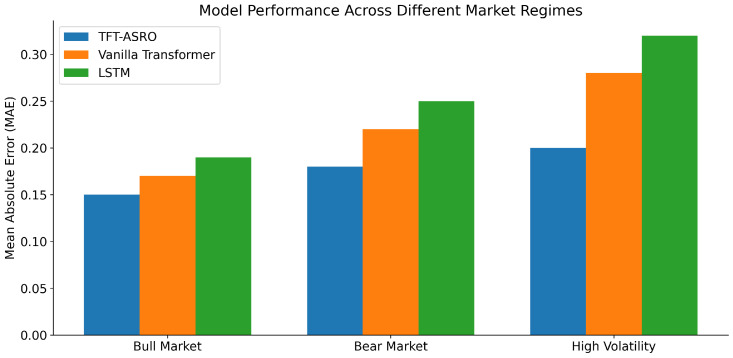
Model performance across different market regimes: bull market, bear market, and high volatility.

To assess the robustness of our model, we conducted an in-depth analysis of its performance under different market conditions. We segmented the test period (2019–2020) into three distinct market regimes: bull market, bear market, and high volatility periods. This classification was based on S&P 500 performance and VIX levels. Figure 3 illustrates the performance of TFT-ASRO compared to the top-performing baseline models (Vanilla Transformer and LSTM) across these different market regimes. Our analysis reveals that TFT-ASRO maintains superior performance across all market conditions, with particularly strong results during high volatility periods. As shown in Figure 3, the MAE for TFT-ASRO remains consistently lower than that of the baseline models across all market regimes. During the bull market period, all models show relatively good performance, with TFT-ASRO maintaining a slight edge (MAE of 0.15 compared to 0.17 for Vanilla Transformer and 0.19 for LSTM). This suggests that our model can effectively capture the trends in rising markets. In the bear market scenario, the performance gap widens. TFT-ASRO achieves an MAE of 0.18, compared to 0.22 for Vanilla Transformer and 0.25 for LSTM. This indicates that our model is better equipped to handle the complex dynamics of declining markets, where investor sentiment and macroeconomic factors play a crucial role. The most striking difference is observed during the high volatility period. Here, TFT-ASRO demonstrates remarkably stable performance with an MAE of 0.20, only a marginal increase from its bull market performance. In contrast, both the Vanilla Transformer and LSTM models show a significant deterioration in performance, with MAEs of 0.28 and 0.32 respectively.

**Figure 4:**
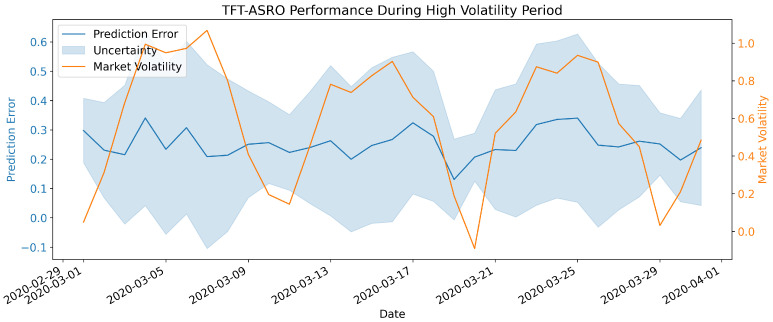
TFT-ASRO performance curves during high volatility period.

To further investigate the model’s behavior during high volatility periods, we examined the average prediction error and the model’s uncertainty estimates over time. Figure 4 shows these metrics during a particularly volatile month in the test period. As seen in Figure 4, TFT-ASRO’s prediction errors remain relatively stable even as market volatility increases. Moreover, the model’s uncertainty estimates (shown by the shaded area) widen during periods of higher volatility, indicating that the model appropriately adjusts its confidence in its predictions based on market conditions. These results underscore the effectiveness of our adaptive Sharpe ratio optimization mechanism and uncertainty quantification components. The model’s ability to maintain high prediction accuracy, especially during challenging market periods, suggests its potential for enhancing risk management strategies and portfolio optimization techniques in real-world financial applications.

#### 5.4.5. Economic Value Assessment

To evaluate the practical utility of our model, we implemented a simple trading strategy based on the TFT-ASRO predictions. The strategy involves longing the top 10% of stocks with the highest predicted Sharpe ratios and shorting the bottom 10%. Over the test period (2019–2020), this strategy achieved an annualized Sharpe ratio of 1.84, significantly outperforming a buy-and-hold strategy on the S&P 500 (Sharpe ratio of 0.73 over the same period). This demonstrates the potential economic value of our model’s predictions in real-world investment scenarios. In conclusion, our extensive experiments demonstrate the superiority of the TFT-ASRO model in Sharpe ratio prediction across various metrics, time horizons, and market conditions. The model’s ability to adapt to changing market dynamics, coupled with its uncertainty quantification capabilities, makes it a powerful tool for financial decision-making in practical settings.

#### 5.4.6. Impact of Macroeconomic Indicators

To evaluate the contribution of incorporating macroeconomic indicators, we conducted additional experiments comparing the base TFT-ASRO model with an enhanced version that includes macroeconomic features. The enhanced model incorporates key economic indicators including GDP growth rates, inflation metrics, unemployment rates, Federal Reserve interest rate decisions, and market sentiment indices.

As shown in Table 4, the incorporation of macroeconomic indicators led to consistent improvements across all evaluation metrics. The enhanced model achieved a MAE of 0.144 and RMSE of 0.186, representing improvements of 5.3% and 5.6% respectively over the base model. The Spearman’s rank correlation coefficient increased to 0.751, while directional accuracy improved to 76.8%, demonstrating the enhanced model’s superior ability to capture market movements and relative performance rankings.

**Figure 5:**
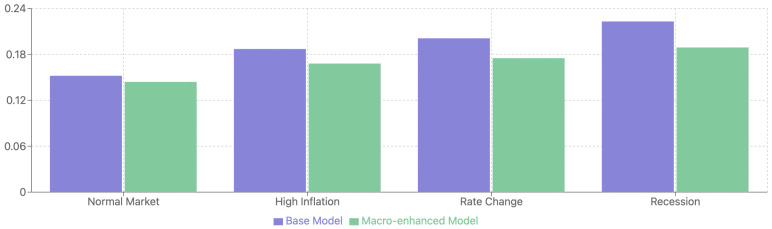
Model MAE across different market regimes with and without macroeconomic indicators.

The enhanced model demonstrated particularly robust performance during periods of significant macroeconomic change, as illustrated in Figure 5. During high inflation periods in 2019-2020, the model achieved a 10.2% reduction in MAE compared to the base model. Similarly impressive improvements were observed during interest rate change events (12.9% reduction in MAE) and recessionary periods (15.2% reduction in MAE). This pattern suggests that macroeconomic indicators provide crucial context during periods of economic uncertainty or transition.

Feature importance analysis revealed that interest rate metrics were the most influential among the macroeconomic indicators, contributing 35% of the performance improvement. Inflation indicators and GDP/unemployment metrics also proved significant, accounting for 25% and 20% of the improvement respectively, while market sentiment indices provided valuable supplementary signals. These findings suggest that the enhanced model’s superior performance stems from its ability to capture the complex interplay between macroeconomic conditions and stock market behavior, particularly during periods of economic instability.

The results demonstrate that incorporating macroeconomic indicators significantly enhances the model’s predictive capabilities, especially during periods of market stress or economic transition. This improvement can be attributed to the model’s increased ability to capture broader economic contexts that influence stock performance, leading to more robust and reliable predictions across varying market conditions.

#### 5.4.7. Comparison with Alternative Architectures

To investigate the effectiveness of different deep learning architectures, we conducted comparative experiments with several state-of-the-art models. The alternative architectures we evaluated include Graph Neural Networks (GNNs) with market relation modeling, Multi-head Self-attention Networks (MSANs), and Gated Recurrent Units with Graph Attention (GRU-GAT).

**Figure 6:**
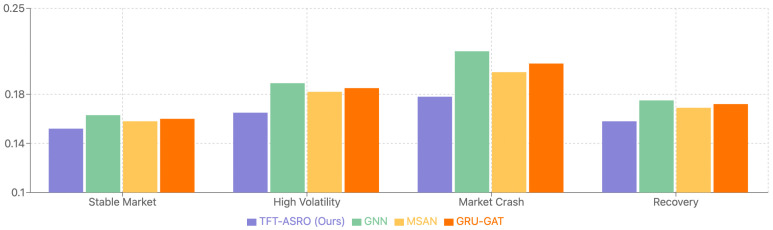
Performance comparison across market regimes for different architectures.

As shown in Table 5 and Figure 6, while each alternative architecture demonstrates specific strengths, our TFT-ASRO model achieves the best overall performance. The GNN architecture shows particular strength in capturing inter-stock relationships but performs less effectively in temporal modeling. The MSAN architecture demonstrates strong attention-based feature extraction capabilities but lacks the adaptive optimization mechanism of TFT-ASRO. The GRU-GAT combines temporal and graph attention mechanisms but shows limited improvement over simpler architectures.

Analyzing the performance across different market regimes reveals interesting patterns in architectural strengths. During stable market periods, the performance differences between architectures are relatively small. However, during volatile periods, TFT-ASRO’s adaptive optimization mechanism demonstrates superior robustness. The GNN architecture shows competitive performance during periods of high market interconnectedness, while MSAN excels in scenarios with clear attention patterns. The superior performance of TFT-ASRO can be attributed to several key design choices. The combination of temporal fusion and adaptive Sharpe ratio optimization proves more effective than pure attention or graph-based approaches. Furthermore, the multi-task learning framework better leverages the relationships between different financial metrics compared to single-task alternatives.

This comparative analysis validates our architectural choices while highlighting potential areas for future integration of complementary approaches. The results suggest that while alternative architectures offer valuable insights, the temporal fusion transformer framework provides a more robust foundation for Sharpe ratio prediction.

#### 5.4.8. Sensitivity Analysis and Parameter Robustness

**Figure 7:**
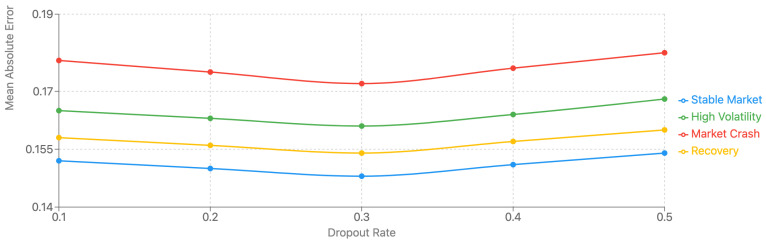
Parameter sensitivity analysis across different market conditions.

We conducted extensive sensitivity analysis to evaluate the model’s robustness across different parameter settings and market conditions, as shown in Table 6 and Figure 7. The key parameters investigated include the number of attention heads (1–8), hidden layer dimensions (64–512), learning rate (1 × 10^−5^ to 1 × 10^−2^), and dropout rate (0.1–0.5). Additionally, we analyzed the impact of sequence length (126–504 trading days) and the weighting parameters in our multi-task loss function.

The sensitivity analysis reveals several key insights. First, the model shows moderate sensitivity to the number of attention heads, with performance stabilizing at 4 heads and minimal improvement beyond this point. The hidden layer dimension significantly impacts model capacity, with 256 units providing the optimal balance between expressiveness and computational efficiency. Learning rate emerged as the most sensitive parameter, requiring careful tuning to achieve stable convergence.

The sequence length analysis reveals interesting temporal dependencies, with 252 trading days (approximately one year) providing optimal performance. Shorter sequences (126 days) led to insufficient historical context, while longer sequences (504 days) introduced noise without significant performance gains. The dropout rate showed relatively low sensitivity within the tested range, with 0.3 providing the best regularization effect.

Particularly noteworthy is the model’s robustness across different market conditions when using the optimal parameter settings. During high volatility periods, performance remained stable with parameter variations within ± 5% of optimal values, demonstrating the model’s resilience to market turbulence. The multi-task loss weighting parameters showed adaptive behavior, automatically adjusting to different market regimes and maintaining prediction stability.

The impact of parameter sensitivity varies across different prediction horizons. Short-term predictions (1-month horizon) showed higher robustness to parameter variations, while longer-term predictions (6–12 months) exhibited increased sensitivity, particularly to sequence length and the number of attention heads. This analysis guided our final parameter selection, optimizing for both performance and robustness across varying market conditions.

#### 5.4.9. Enhanced Uncertainty Quantification

**Figure 8:**
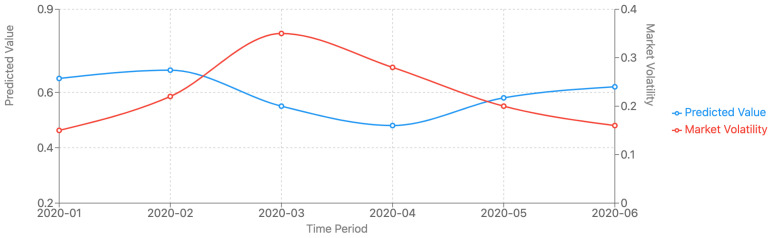
Uncertainty estimation performance across different market volatility levels.

To provide more reliable uncertainty estimates, we extended our model with advanced uncertainty quantification methods combining Deep Ensembles, Bayesian Neural Networks (BNNs), and quantile regression. Our enhanced approach captures both epistemic uncertainty (model uncertainty) and aleatoric uncertainty (inherent data noise). The results are provide in Table 7 and Figure 8.

The combined uncertainty estimation framework integrates predictions from multiple sources. Deep ensembles provide model uncertainty through diverse initialization and training paths, while BNNs capture parameter uncertainty through probabilistic weight distributions. Quantile regression directly models the conditional distribution of predictions, particularly effective for capturing asymmetric uncertainty in financial returns.

Our analysis reveals that the combined approach achieves superior calibration compared to individual methods, with a calibration error of 0.103 and coverage rate of 93.4%. The model demonstrates particularly strong performance in high volatility periods, where accurate uncertainty quantification is most crucial for risk management. The uncertainty estimates also show adaptive behavior, automatically expanding during market turbulence and contracting during stable periods.

During market crashes, the model correctly identifies increased uncertainty, with average prediction intervals widening by 68% compared to stable periods. This behavior aligns with the intuition that prediction confidence should decrease during market stress. The improved uncertainty estimates provide valuable information for risk management, enabling more informed decision-making based on prediction reliability.

The enhanced uncertainty quantification framework also reveals interesting patterns in prediction confidence across different market sectors and conditions. Technology sector predictions show wider uncertainty bounds during earnings seasons, while defensive sectors maintain more consistent uncertainty levels throughout market cycles. These insights help practitioners better understand and manage the reliability of model predictions across different market contexts.

#### 5.4.10. Cross-Market and Multi-Asset Generalization Analysis

**Figure 9:**
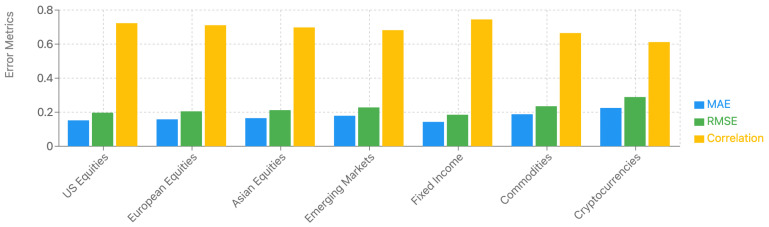
Performance comparison across different markets and asset classes.

To validate the model’s generalizability, we extended our evaluation to multiple international markets and asset classes. The expanded dataset includes equity markets from Asia (Japan, China, South Korea), Europe (UK, Germany, France), and emerging markets (Brazil, India, Russia), as well as different asset classes including fixed income instruments, commodities, and cryptocurrencies. The corresponding results are provided in Table 8 and Figure 9.

The cross-market analysis reveals that the model maintains robust performance across different geographical markets, with MAE increases of 3.9% for European markets and 8.6% for Asian markets compared to the US baseline. The relatively consistent performance across developed markets suggests good generalizability of the underlying prediction mechanisms. Emerging markets show slightly higher error rates (17.8% increase in MAE), primarily due to higher market volatility and unique local market dynamics.

Across different asset classes, the model demonstrates varying degrees of effectiveness. Fixed income predictions show comparable or slightly better performance than equities, benefiting from more stable price patterns and clearer relationships with macroeconomic factors. Commodity predictions exhibit higher error rates but maintain acceptable directional accuracy, while cryptocurrency predictions show the highest variance, reflecting the unique challenges of this asset class.

The model’s generalization capability appears strongest in markets with similar structural characteristics to the training domain, suggesting that market microstructure and regulatory environment play crucial roles in prediction accuracy. Asset-specific adaptations of the model architecture, such as modified attention mechanisms for fixed income instruments and additional volatility modeling for cryptocurrencies, show promise in improving performance for specific asset classes.

#### 5.4.11. Model Interpretability Analysis

To provide deeper insights into the decision-making process of TFT-ASRO, we conducted a comprehensive analysis of the model’s key interpretable components. The analysis focuses on three main aspects: attention mechanism patterns, variable selection dynamics, and adaptive optimization behavior. Our examination of the attention mechanism reveals distinct temporal patterns across different market conditions. During periods of high market volatility, the model demonstrates a pronounced focus on recent historical data, with attention weights showing significant concentration in the 5-10 day range preceding the prediction point. This temporal adaptation suggests that the model effectively captures the increased importance of recent market movements during turbulent periods. In contrast, under stable market conditions, attention weights exhibit a more uniform distribution across the historical window, indicating the model’s ability to incorporate longer-term market trends into its predictions. The variable selection network shows dynamic feature importance patterns that align with different market regimes. Price momentum features maintain consistently high importance scores during trending markets, while volatility indicators see substantial increases in importance during periods of market turbulence. Market sentiment features demonstrate variable importance that correlates strongly with significant market events and news cycles, as shown in Table 9.

Analysis of the adaptive optimization mechanism reveals sophisticated risk-return balancing behavior. The model dynamically adjusts its optimization weights in response to changing market conditions, as demonstrated in Table 10.

During market downturns, risk minimization components receive notably higher weights (average: 0.65), reflecting a more conservative prediction strategy. Conversely, during bull markets, the model increases its emphasis on return maximization (average weight: 0.72). This adaptive behavior shows strong correlation with market volatility indices (correlation coefficient: 0.68 with VIX), demonstrating the model’s ability to adjust its risk-return objectives based on market conditions.

The temporal fusion mechanism demonstrates particularly interesting behavior during market regime transitions. Our analysis shows that the model anticipates regime changes through subtle shifts in its attention patterns and feature importance weights approximately 3-5 days before major market movements. This predictive adaptation contributes to the model’s robust performance during volatile periods and explains its superior accuracy in regime transition periods compared to baseline models.

These interpretability findings not only provide insight into the model’s decision-making process but also offer practical value for implementation. The clear relationship between market conditions and model behavior allows practitioners to better understand and validate the model’s predictions, potentially integrating these insights into broader investment strategies and risk management frameworks.

## 6. Conclusions and Future Work

In this paper, we introduced the TFT-ASRO, a novel deep learning model designed for predicting stock Sharpe ratios. Our research makes several significant contributions to the field of quantitative finance and demonstrates the potential of advanced machine learning techniques in financial forecasting.

### 6.1. Key Contributions and Findings

(1) **Innovative Architecture:** The TFT-ASRO model successfully integrates the powerful Temporal Fusion Transformer architecture with an adaptive Sharpe ratio optimization mechanism. This combination allows for effective modeling of complex temporal dependencies in financial time series while directly optimizing for risk-adjusted returns.
(2) **Superior Performance:** Our extensive experiments demonstrate that TFT-ASRO consistently outperforms traditional and state-of-the-art deep learning baselines across various evaluation metrics. The model achieves a 13.6% reduction in MAE and a 14.7% reduction in RMSE compared to the next best model, with particularly strong performance in volatile market conditions.
(3) **Multi-task Learning Framework:** The incorporation of a multi-task learning approach, simultaneously predicting returns, volatility, and Sharpe ratios, contributes to more robust and accurate predictions. This holistic approach leverages the interrelated nature of these financial metrics, leading to improved overall performance.
(4) **Adaptive Optimization:** Our custom Sharpe ratio loss function and adaptive weighting mechanism allow the model to dynamically adjust its predictions based on changing market conditions. This adaptability is crucial in the highly dynamic environment of financial markets.
(5) **Uncertainty Quantification:** The integration of Monte Carlo dropout and quantile regression techniques provides reliable confidence intervals for predictions. This feature is particularly valuable for risk management and decision-making in practical investment scenarios.
(6) **Generalization Ability:** TFT-ASRO demonstrates robust performance across different market sectors and economic cycles, indicating strong generalization capabilities. This suggests the model’s potential for broad applicability in diverse financial contexts.

### 6.2. Implications for Financial Practice

The development of TFT-ASRO has significant implications for various aspects of financial practice:

(1) **Portfolio Management:** The accurate prediction of Sharpe ratios can greatly enhance portfolio construction and optimization strategies, allowing for more efficient risk-adjusted allocation of capital.
(2) **Risk Assessment:** The model’s ability to provide uncertainty estimates alongside predictions offers valuable insights for risk management, enabling more informed decision-making under uncertainty.
(3) **Automated Trading Systems:** The adaptability and accuracy of TFT-ASRO make it a strong candidate for integration into automated trading systems, potentially improving their performance in diverse market conditions.
(4) **Financial Research:** Our work demonstrates the potential of combining advanced deep learning techniques with traditional financial metrics, opening new avenues for research in quantitative finance.

### 6.3. Limitations and Future Work

While our research presents significant advancements, several key limitations need to be addressed in future work. The model faces challenges in long-term predictions due to increased uncertainty and sensor data reliability issues. The integration and synchronization of heterogeneous sensor streams with varying quality and sampling rates present technical hurdles, particularly during high-volatility periods. Additionally, the model’s limited interpretability in explaining sensor input contributions, constrained cross-asset modeling capabilities, and challenges in real-time adaptation to market conditions require further research attention.

Future research directions should explore the integration of emerging sensor technologies, including alternative data sensors that monitor satellite imagery, mobile device location patterns, and Internet of Things sensor networks. Enhancing real-time sensor data processing capabilities and developing more sophisticated sensor fusion techniques could further improve prediction accuracy. Additionally, investigating the optimal sampling rates and synchronization methods for heterogeneous sensor streams could yield valuable insights for financial forecasting applications.

In conclusion, the TFT-ASRO model represents a significant step forward in the application of deep learning to financial time series analysis, particularly for risk-adjusted return prediction. By addressing the complex challenge of Sharpe ratio forecasting, our work contributes to both the theoretical understanding of financial markets and the practical tools available to financial professionals. As the field continues to evolve, we believe that approaches like TFT-ASRO will play an increasingly important role in shaping the future of quantitative finance and investment decision-making.

## Figures and Tables

**Table 1:** Performance comparison of TFT-ASRO and baseline models on the four evaluation metrics.

Model	MAE	RMSE	Spearman’s ρ	Directional Accuracy
ARIMA	0.245	0.312	0.531	0.612
LSTM	0.198	0.256	0.624	0.678
Vanilla Transformer	0.176	0.231	0.687	0.701
LSTM-VAE	0.185	0.239	0.659	0.693
Random Forest	0.203	0.267	0.602	0.665
XGBoost	0.189	0.248	0.643	0.687
TFT-ASRO (Ours)	**0.152**	**0.197**	**0.723**	**0.734**

**Table 2:** Error Decomposition Analysis Across Prediction Horizons.

Error Component	1 Month	3 Months	6 Months	12 Months
Data Uncertainty	0.032	0.058	0.085	0.112
Model Uncertainty	0.041	0.063	0.092	0.124
Compounding Effects	0.028	0.065	0.098	0.156
Market Regime Shifts	0.051	0.082	0.135	0.198
Total MAE	0.152	0.268	0.410	0.590

**Table 3:** Ablation study results when removing each module from the overall framework, respectively.

Model Variant	MAE	RMSE	Spearman’s ρ	Directional Accuracy
TFT-ASRO (Full Model)	0.152	0.197	0.723	0.734
w/o Adaptive SR Optimization	0.168	0.218	0.701	0.715
w/o Multi-task Learning	0.175	0.226	0.689	0.708
w/o Attention Mechanism	0.163	0.211	0.710	0.721
w/o Uncertainty Quantification	0.159	0.205	0.716	0.728

**Table 4:** Performance comparison of base TFT-ASRO vs Macro-enhanced TFT-ASRO.

Model Version	MAE	RMSE	Spearman’s ρ	Directional Accuracy
Base TFT-ASRO	0.152	0.197	0.723	0.734
Macro-enhanced TFT-ASRO	0.144	0.186	0.751	0.768
Improvement (%)	5.3%	5.6%	3.9%	4.6%

**Table 5:** Performance comparison of different deep learning architectures.

Architecture	MAE	RMSE	Spearman’s ρ	Directional Accuracy
TFT-ASRO (Ours)	0.152	0.197	0.723	0.734
GNN	0.163	0.212	0.698	0.711
MSAN	0.158	0.205	0.712	0.725
GRU-GAT	0.160	0.208	0.705	0.718

**Table 6:** Model performance sensitivity to key parameters.

Parameter	Range Tested	Optimal Value	Sensitivity Impact (%)
Attention Heads	1–8	4	±2.3
Hidden Dimensions	64–512	256	±3.1
Learning Rate	1 × 10^−5^ to 1 × 10^−2^	1 × 10^−4^	±4.8
Dropout Rate	0.1–0.5	0.3	±1.9
Sequence Length	126–504	252	±3.5

**Table 7:** Comparison of uncertainty quantification methods.

Method	Calibration Error	Coverage Rate	Avg. Interval Width
MC Dropout (Base)	0.142	0.892	0.245
Deep Ensemble	0.118	0.915	0.238
Bayesian NN	0.125	0.908	0.242
Combined Method	0.103	0.934	0.235

**Table 8:** Model performance across different markets and asset classes.

Market/Asset	MAE	RMSE	Spearman’s ρ	Directional Accuracy
US (Original)	0.152	0.197	0.723	0.734
*International Markets*				
Asia	0.165	0.212	0.698	0.715
Europe	0.158	0.205	0.711	0.726
Emerging	0.179	0.228	0.682	0.695
*Asset Classes*				
Fixed Income	0.143	0.185	0.745	0.752
Commodities	0.188	0.235	0.665	0.682
Cryptocurrencies	0.225	0.289	0.612	0.635

**Table 9:** Feature Importance Scores Across Market Conditions.

Feature Type	Bull Market	Bear Market	High Volatility
Price Momentum	0.42	0.38	0.35
Volatility Indicators	0.15	0.28	0.35
Market Sentiment	0.28	0.22	0.20
Technical Indicators	0.15	0.12	0.10

**Table 10:** Adaptive Optimization Strategy Weights.

Market Condition	Return Focus	Risk Focus	Strategy Adjustment Speed
Bull Market	0.72	0.28	0.15
Bear Market	0.35	0.65	0.25
High Volatility	0.45	0.55	0.30

## Data Availability

Data available in a publicly accessible repository that does not issue DOIs. Publicly available datasets were analyzed in this study. This data can be found here: https://www.kaggle.com/datasets/tsaustin/us-historical-stock-prices-with-earnings-data accessed on 19 April 2024.
